# Quarantine due to the COVID-19 pandemic from the perspective of adolescents: the crucial role of technology

**DOI:** 10.1186/s13052-021-00997-7

**Published:** 2021-02-22

**Authors:** Giuseppina Salzano, Stefano Passanisi, Francesco Pira, Lacrima Sorrenti, Giuseppa La Monica, Giovanni Battista Pajno, Maria Pecoraro, Fortunato Lombardo

**Affiliations:** 1 Department of Human Pathology in Adult and Developmental Age “Gaetano Barresi”, University of Messina, Via Consolare Valeria 1, 98124 Messina, Italy; 2 Department of Ancient and Modern Civilizations, University of Messina, Messina, Italy

**Keywords:** Adolescence, Lock-down, Outbreak, SARS-CoV-2, Self-isolation, Social media

## Abstract

**Background:**

The year 2020 will be remembered as the “year of the COVID-19 pandemic”. The world population had to familiarize themselves with words as swabs, personal protective equipment, pandemic. To curb the wave of the pandemic, almost all the countries imposed self-isolation and social distancing. We conducted a web-based survey to investigate the behavioural responses during the quarantine due to the COVID-19 pandemic.

**Methods:**

Participants were 1860 youth aged 12–18 years attending lower secondary schools and upper secondary schools. Data were collected on demographic characteristics, lifestyle changes during the quarantine period, and the psychological impact of the lock-down on adolescents’ life.

**Results:**

Most adolescents experienced feelings of fear, discouragement, and anxiety which strongly affected the approach to their daily lifestyles. Most of the surveyed subjects reported having used this period to acquire new skills and to practice physical activities at home. The use of technology was predominant both for recreational activities and educational purposes.

**Conclusions:**

Despite the strong psychological impact of the quarantine, adolescents showed good levels of resilience. Technology played a crucial role during the quarantine for young subjects who have increased the daily use of technological devices to stay connected with the rest of the world.

**Supplementary Information:**

The online version contains supplementary material available at 10.1186/s13052-021-00997-7.

## Background

Since the beginning of 2020, health authorities and government around the world are fighting the dramatic battle against the 2019 coronavirus disease (COVID-19). Within few weeks of the first cases being reported in China, severe acute respiratory syndrome coronavirus 2 (SARS-CoV-2) infection spread worldwide, and a pandemic was finally declared by the World Health Organization (WHO) in March 11th, 2020 [1]. To curb the wave of the pandemic, almost all the countries undertook containment strategies. In most cases, these measures resulted in strict governmental decrees that imposed self-isolation and social distancing, the so-called quarantine or “lock-down” period [2]. Italy, which was one of the most affected countries by disease in Europe, set to quarantine the entire country from March 9th to May 3rd, 2020 for a total of 55 days**.** These restrictive strategies led to a gradual and constant reduction of new cases of infection [3–5]. Nevertheless, the quarantine period had a strong psychological impact on the population. People were forced to radically change their daily lifestyles and were at high risk of developing feelings of fear, discouragement, anxiety, and depression [6]. This underestimated consequence of the COVID-19 pandemic was mostly noticed in some risk groups such as older people, individuals suffering from chronic disease, children, and adolescents [7–10]. Particularly, adolescence is a crucial period of human life that is characterized by the physiological evolution of somatic and neuroendocrine characteristics associated with psychological and behavioural modifications. In this period of life, the subject experiences a process of growth, the development of his own personality, and the discovery of himself. Adolescents aim to establish a cognitive and emotional connection with the social environment and setting. One of the main objectives of adolescents is the achievement of autonomy, which requires an inner journey through certainty and confusion, euphoria and anxiety, satisfaction and complaining. Therefore, adolescence is considered a tricky transition period during which rebellion and lawlessness are frequently observed [11]. Respecting safer-at-home order and ban on any outdoor activities was not easy for young subjects who are used to daily sharing face to face experiences, feelings, emotions.

This study aimed to investigate the behavioural responses during the quarantine due to the COVID-19 pandemic in a large cohort of Italian adolescents.

## Methods

We conducted a cross-sectional survey based on an on-line questionnaire from April 23 to May 3, 2020. Survey participants were subjects aged 12–18 years attending lower secondary schools and upper secondary schools. The online link for the questionnaire was sent to the headteachers of 15 schools of Sicily, in southern Italy, who invited all their students to participate. Written informed consent through on-line form was obtained from all the participants. The study research was conducted in accordance with the Helsinki declaration. The questionnaire included seventeen questions focusing on demographic characteristics (e.g. age, gender, type of school attended), lifestyle changes during the quarantine period (variations of eating habits, acquisition of new skills, changes in the sleep-weak rhythm, time spent on indoor physical activities, the use of technological devices such as personal computers, smartphones, tablets). The participants were asked if they experienced feelings of fear, discouragement, and anxiety during the lock-down, and to mention who or what they mostly lack. Finally, they were invited to quantify the psychological impact of the self-isolation and social distancing according to three response levels: no impact, poor impact, extreme impact.

An English translation of the full Italian questionnaire is available as supplementary online material.

Demographic patients’ characteristics and the results of the questionnaire were statistically analyzed. The numerical data were expressed as mean and standard deviation and the categorical variables as absolute frequencies and percentages.

## Results

Our study population included 1860 adolescents. The mean age of the surveyed subjects was 16 ± 1.9 years, with a prevalence of female gender (61.7%). Most of the participants (88%) attended upper secondary school. Self-isolation and social distancing strongly influenced the everyday life of the majority of adolescents (70.2%), whereas only 6.8% of the individuals declared they were not affected in their approach to their daily lifestyles. Feelings such as fear, discouragement, and anxiety were widely present. The surveyed subjects mainly suffered from the lack of their friends, classmates, and partners. To visit relatives, to go to school, and to do outdoor sports were all missing activities during this quarantine period. Some participants declared they suffered from a lack of enjoyment and freedom. Most adolescents (81.5%) modified their sleep/wake rhythm. Almost two-thirds of the surveyed subjects (64.5%) took advantage of this period to acquire new skills such as cooking, reading books, learning to do it yourself activities, play an instrumental or a foreign language. Interestingly, 47.5% of adolescents declared no variations in their eating habits. Moreover, 25.6% of the surveyed individuals reported a more balanced diet, whereas 26.8% of subjects declared their eating habits worsened. Despite Italian governmental decrees prohibited outdoor sports, 84.5% of the survey participants regularly practiced physical activities at home. Particularly, most of them spent from 1 to 3 h a week to do sport. The use of technology was predominant both for recreational activities (communications, games, videos) and for educational purposes (scholar, musical, and sportive activities). The average time spent on technology was mostly more than 6 h a day for educational purposes and 4–6 h a day for recreational activities. Almost all the subjects declared having a social profile (i.e. Instagram, Facebook, Tik Tok, Twitter, Snapchat, Ask.fm). Smartphones were mainly used to message, chat, or video-chat with other people, and to browse the web. The average number of sent/received text messages or chat messages were over 100 a day.

A detailed overview of the results of the web-based survey is available in Table 1.

**Table 1:** Overview of the results of web-based survey

N°	1860
**Age** (ys)	16.0 ± 1.9
**Gender**	
Female	1147 (61.7%)
Male	713 (38.3%)
**Attended school**	
Lower secondary schools	224 (12%)
Upper secondary schools	1636 (88%)
**Feelings of fear, discouragement, anxiety**	
Yes	1123 (60.4%)
No	737 (39.6%)
**The influence of self-isolation on the approach to everyday life**	
Relevant impact	1305 (70.2%)
Little impact	428 (23.0%)
No impact	127 (6.8%)
**Main lack due to quarantine**	
Friends, schoolmates, partners	684 (36.8)
Families	276 (14.8%)
Outdoor sport	246 (13.2%)
School	262 (14.1%)
Freedom	250 (13.4%)
Enjoying life	142 (7.7%)
**Modification of sleep-weak rhythm**	
Yes	1516 (81.5%)
No	344 (18.5%)
**New skills acquired**	
Cooking	631 (33.9%)
Reading books	238 (12.8%)
Do it yourself activities	196 (10.6%)
Learning to play an instrument	60 (3.2%)
Learning a new language	59 (3.2%)
Others (drawing, painting, etc)	14 (0.8%)
None	660 (35.5%)
**Variations in eating habits**	
More balanced diet	477 (25.6%)
Less balanced diet	499 (26.8%)
No differences	884 (47.5%)
**Time spent on physical activity at home**	
< 1 h a week	355 (19.1%)
1–3 h a week	559 (30.1%)
4–6 h a week	324 (17.4%)
> 6 h a week	333 (17.9%)
Not practiced	289 (15.5%)
**Time spent on technology (desktop computers, smartphones, tablets) for educational purposes**	
< 3 h a day	295 (15.9%)
4–6 h a day	640 (34.4%)
> 6 h a day	925 (49.7%)
Not used	/
**Time spent on technology (desktop computers, smartphones, tablets) for recreational activities**	
< 3 h a day	573 (30.8%)
4–6 h a day	765 (41.1%)
> 6 h a day	513 (27.6%)
Not used	9 (0.5%)
**Owning a personal computer**	
Yes	1640 (88.2%)
No	220 (11.8%)
**Owning a smartphone**	
Yes	1791 (96.3%)
No	69 (3.7%)
**Social profile (Instagram, Facebook, Tik Tok, Twitter, Ask.fm)**	
Yes	1835 (98.7%)
No	25 (1.3%)
**Way to use smartphone**	
Messages, chat or video-chat	860 (46.2%)
Browse the web	314 (16.9%)
Talk on the phone	76 (4.1%)
Download/listen music	35 (1.9%)
Download/watch video	40 (2.2%)
All of the above	535 (28.7%)
**Number of text messages and/or chat messages**	
0–10 a day	29 (1.6%)
11–30 a day	88 (4.7%)
31–50 a day	206 (11.1%)
51–100 a day	392 (21.1%)
> 100 a day	1145 (61.6%)

## Discussion

Although the exact rate of pediatric patients affected by COVID-19 worldwide is not available thus far, children and adolescents seem to be less infected [12]. According to COVID-19 integrated surveillance data in Italy, only 3.1% of patients diagnosed with SARS-CoV-2 infection were aged 0–18 years [13]. However, it is well known that children and adolescents who are quarantined during pandemic diseases had a high risk of acute stress disorder. A preliminary study conducted in the Shaanxi province during the COVID-19 epidemic showed that clinging, inattention, and irritability were the most severe psychological conditions demonstrated by children and adolescents [9]. Our survey confirmed that the majority of adolescents experienced feelings of fear, discouragement, and anxiety due to the lock-down, which strongly affected the approach to their daily lifestyles.

Technology has played a crucial role during the quarantine for young subjects who have increased the daily use of technological devices to stay connected with the rest of the world. Particularly, social media platforms have become fundamental for maintaining and enhancing socialization. These interactive media platforms have been constantly used to maintain friendship and emotional connection. Nowadays, the real-life of adolescents is closely related to their “online environments”, and social media have become an integral part of critical adolescent developmental tasks [14]. These online environments reflect, complement, and reinforce well-understood psychological mechanisms, such as social comparison, self-disclosure, and impression management [15]. Social media tools also allow adolescents to enhance individual and collective creativity through the sharing of artistic and musical activities, the creation of blogs, podcasts, and videos [16]. In some cases, the frequent use of social networks can mitigate negative experiences such as psychosocial maladjustment and relationship difficulties and allows them to remove fears and insecurities [17]. The use of social media tools can also facilitate self-esteem increase, identity exploration, aspirational development, and it provides adolescents the opportunity to explore knowledge and establish new friendships [18]. Our survey showed the crucial role of friendship during the adolescence age. Face-to-face contact with close friends has been the main lack due to restrictive laws among the surveyed adolescents. To obviate this lack, text messages, video-chats, and online contacts have become more frequent during the lock-down period. Therefore, face-to-face communication has been well replaced by virtual and interactive contact characterized by close friendships and emotional relationships [19].

Despite the strong psychological impact of the quarantine, adolescents have shown good levels of resilience. Most of the surveyed subjects reported having used this period to acquire new skills. This positive response to the social emergency is related to the development of functional empowerment which allows reaching a satisfactory level of interior maturity. Interestingly, most of the subjects spent at least 1–3 h a week for indoor physical activity. This finding is accordingly with another Italian survey that demonstrated an increase in training frequency [20].

Sleep-wake habits were radically changed during the lock-down, as demonstrated by the majority of the surveyed adolescents who declared modification in their sleep/weak rhythm. This finding is in agreement with a French cross-sectional study that reported a high rate of trouble sleeping, particularly among young people [21]. However, it should not be overlooked that sleep disturbances could also be related to the exaggerated use of technological devices at night. “Vamping” is a term that refers to some young people’s practice to stay online all night during which they text, share videos, and post selfies on social media. Previous studies have already demonstrated that alterations in neurophysiological mechanisms of sleep and circadian rhythm affect tech-addicted adolescents who are at greater risk for bad school performance and loss of self-control [18]. Use of social media out of all proportion may also have other negative effects. It is widely accepted that virtual socialization could cause cyberbullying, sexting, and various psychological disorders such as depression and anxiety that adversely affect the development of the adolescent’s identity [18].

Regarding the dietary habits, almost half of adolescents have not reported any modifications. Otherwise, 25.6% of those interviewed declared a more balanced diet. We suppose that abstention from school and peer relationships out of school, has helped to maintain a healthy diet since the consumption of savory snacks, snacks, processed meat, carbonated and sugary drinks decreased [20]. On the contrary, the consumption of healthy homemade food prevailed.

Finally, the lockdown period allowed to strengthen the relationship between adolescents and their parents at home in most cases. The critical role of the family in adolescent neuropsychological development is well documented. The relationship between adolescents and their families is based on two opposite aspects: preserving a close bond with their family and the need to achieve autonomy. The quality of family relationships is related to the way how adolescents cope with core developmental tasks and the transition to adulthood [22]. Consequently, dialogue and mutual listening are crucial in the parent-child relationship as they allow to create emotional sharing and ensure the adolescent’s growth [23]. Spending a lot of time at home has undoubtedly encouraged adolescents to have daily, constructive interactions with their parents which allowed them to gain awareness of their maturity process.

## Conclusions

Quarantine due to the COVID-19 pandemic was something new for the most of population worldwide. Adolescents were strongly influenced by self-isolation and social distancing. Technology, and particularly social media, were fundamental to allow youth to overcome this stressful period and to limit psychological adverse events related to the lock-down.

## Supplementary Information

**Additional file 1.** Questionnaire: quarantine due to COVID-19 from the perspective of adolescents.

## Data Availability

An English translation of the full Italian questionnaire is available as supplementary online material.

## Abbreviations

COVID-19: 2019 coronavirus disease
SARS-CoV-2: Severe acute respiratory syndrome coronavirus 2
WHO: World Health Organization
