# Supplementary material for: Quarantine due to the COVID-19 pandemic from the perspective of adolescents: the crucial role of technology
Source: Ital J Pediatr. 2021 Feb 22;47:40. doi: 10.1186/s13052-021-00997-7 (PMC7897888; doi:10.1186/s13052-021-00997-7)
Supplement: Supplementary file 1 — Additional file 1. Questionnaire: quarantine due to COVID-19 from the perspective of adolescents. [file 13052_2021_997_MOESM1_ESM.docx]

**Questionnaire: quarantine due to COVID-19 from the perspective of adolescents**

1. How old are you?

2. Gender

1. Male
2. Female

3. Which school do you attend?

1. Lower secondary school
2. Upper secondary school

4. Did you experience feelings of fear, discouragement or anxiety during this period?

1. Yes
2. No

5. How much self-isolation influenced your everyday life?

1. Extreme influence
2. Poor influence
3. No influence

6. Who or what did you lack most?

1. Friend, schoolmates, boyfriend/girlfriend
2. Families
3. School
4. Outdoor sport activities
5. Freedom
6. Enjoying life

7. Did you observe changes in your sleep-weak rhythm?

1. Yes
2. No

8. Which new skills did you acquire during this period?

1. Cooking
2. Reading books
3. Do it yourself activities
4. Learning to play an instrumental
5. Learning a new language
6. Others (drawing, painting, housecleaning, etc)
7. No new skills acquired

9. Did you practiced physical activity at home?

1. <1 hour a week
2. 1-3 hours a week
3. 4-6 hours a week
4. >6 hours a week
5. No physical activity

10. Did you observe any variations in your eating habits?

1. More balanced diet
2. Less balanced diet
3. No differences

11. How much time did you spend on technology for educational purposes (scholar, musical and sportive activities)?

1. <1 hour a day
2. 1-3 hours a day
3. 4-6 hours a day
4. >6 hours a day
5. Not used for this purpose

12. How much time did you spend on technology for recreational purposes (communications, games, videos)?

1. <1 hour a day
2. 1-3 hours a day
3. 4-6 hours a day
4. >6 hours a day
5. Not used for this purpose

13. Do you have a personal computer?7

1. Yes
2. No

14. Do you have a smartphone?

1. Yes
2. No

15. Did you have a social profile (Facebook, Instagram, Tik Tok, Snapchat, Twitter, Ask.fm)?

1. Yes
2. No

16. How did you mostly use your smartphone?

1. Messaging
2. Browsing the web
3. Talking on the phone
4. Downloading/listening music
5. Downloading/watching videos
6. All of the above

17. How many messages did you send and/or receive?

1. 0-10 messages a day
2. 11-30 messages a day
3. 31-50 messages a day
4. 51-100 messages a day
5. >100 messages a day
